# A Case of LGI1-Antibody Limbic Encephalitis Presenting With Faciobrachial Dystonic Seizures

**DOI:** 10.7759/cureus.108308

**Published:** 2026-05-05

**Authors:** Robert Krahmer, Nikesh R Kapadia

**Affiliations:** 1 Internal Medicine, University of South Florida, Tampa, USA

**Keywords:** altered mental status evaluation, autoimmune encephalitis, autoimmune limbic encephalitis, faciobrachial dystonic seizures (fbds), lgi-1, lgi-1 autoimmune encephalitis, lgi-1 encephalitis, lgi-1 limbic encephalitis, rare cause of altered mental status

## Abstract

Leucine-rich glioma-inactivated 1 limbic encephalitis (LGI1-Ab LE) is a rare cause of altered mental status (AMS). Here we present a case of a 59-year-old male who arrived at the hospital with AMS and several months of abnormal movements. He possessed several risk factors for AMS, including intoxication, possible withdrawal, and electrolyte abnormalities. However, due to his atypical symptoms, a thorough evaluation was performed, and a video-electroencephalogram was positive for faciobrachial dystonic seizures (FBDS), which are pathognomonic for LGI1-Ab LE. FBDS are a specific type of seizure characterized by brief ipsilateral contractions of the arm and face. Results from a lumbar puncture later confirmed the diagnosis, which was positive for LGI-1 antibodies. The patient’s symptoms improved with systemic steroids and lacosamide. LGI1-Ab LE is the second most common type of autoimmune encephalitis and can be challenging to diagnose. This case highlights important clinical features that can aid in early recognition, leading to improved patient outcomes.

## Introduction

Autoimmune encephalitis (AE) comprises a group of antibody-mediated inflammatory diseases that target the central nervous system and present with a wide range of neuropsychiatric symptoms [1]. The clinical presentation of each type of AE is largely determined by the type of antibody produced and the region of the brain affected.

N-methyl-D-aspartate receptor (NMDAR)-antibody encephalitis and leucine-rich glioma-inactivated 1 limbic encephalitis (LGI1-Ab LE) are the two most common types of AE [1]. Some of the other, less common variants include gamma-aminobutyric acid A/B (GABA-A/B) receptor encephalitis, contact-associated protein 2 (CASPR2) antibody encephalitis, and glutamic acid decarboxylase 65 (GAD65) antibody encephalitis [1]. All of which present with unique clinical features, laboratory, and imaging findings.

Limbic encephalitis is a subset of AE caused by antibodies targeting the limbic system, a deep brain region located between the cerebral cortex and brainstem. Some of the limbic system's key components are the hippocampus, mammillary bodies, amygdala, and the medial temporal lobes [2]. The limbic system helps regulate several critical functions, including emotional processing, behavior, memory, and learning [2]. Patients with limbic encephalitis often present with vague complaints, including altered mental status (AMS), cognitive decline, behavior changes, memory deficits, seizures, and abnormal movements [3].

AE is diagnosed by a combination of clinical presentation, serum and cerebrospinal fluid (CSF) studies, imaging findings, and video-electroencephalogram (EEG) results. Due to their nonspecific presentation, these conditions can be difficult to identify; however, they often respond well to therapy, making early diagnosis important for prompt treatment.

## Case presentation

A 59-year-old male with a past medical history of hypertension and cocaine use disorder presented to the emergency department in April of 2025 with AMS and weight loss. For the past two months, he had been having gradually progressive episodes of left facial grimacing with sudden flexion of the left arm. These episodes would last for several seconds and occurred multiple times per day. Additional symptoms included progressive difficulty with attention, short-term memory loss, and behavior changes noted by his wife. Recently, he had been involved in two car accidents where he rear-ended another vehicle and was unable to recall any details about the event afterward. The patient smoked half a pack of cigarettes daily, consumed two drinks of alcohol per week, and used cocaine weekly.

On arrival at the emergency department, his vital signs were normal. His exam showed orientation to person and place but not time. He demonstrated bradyphrenia, a generalized cognitive slowing characterized by delayed responses and inattention. The exam also revealed a flat affect, difficulty with short-term memory, and an inability to perform serial sevens or spell “world” backward. His neurological exam was otherwise normal and without focal deficits. Initial laboratories revealed a moderate hypotonic hyponatremia, normal glucose, normal blood gas analysis, and a mild anemia. A urine drug screen was positive for marijuana and cocaine (Table 1).

**Table 1:** Laboratory evaluation MCV: mean corpuscular volume, pCO2: arterial partial pressure of carbon dioxide, HCO3: bicarbonate, ESR: erythrocyte sedimentation rate, CRP: C-reactive protein, CSF: cerebrospinal fluid, WBC: white blood cell, LGI1-IgG: leucine-rich glioma-inactivated 1 immunoglobulin G

Parameter	Patient value	Reference range	Units
Sodium	127	136-145	mmol/L
Chloride	95	98-107	mmol/L
Serum osmolality	269	280-300	mOsm/kg
Glucose	90	70-110	mg/dL
Hemoglobin	12.2	14.1-18.1	g/dL
MCV	66.8	80-97	fL
pH	7.45	7.35-7.46	-
pCO2	41	35-45	mmHg
HCO3	24	22-29	meq/L
ESR	44	0-20	mm/hr
CRP	0.16	0-0.5	mg/dl
CSF WBC	0	0-4	uL
CSF total protein	30.6	15.0-45.0	mg/dL
CSF LGI1-IgG	Positive	Negative	-
Serum LGI1-IgG	Positive	Negative	-

**Figure 1:**
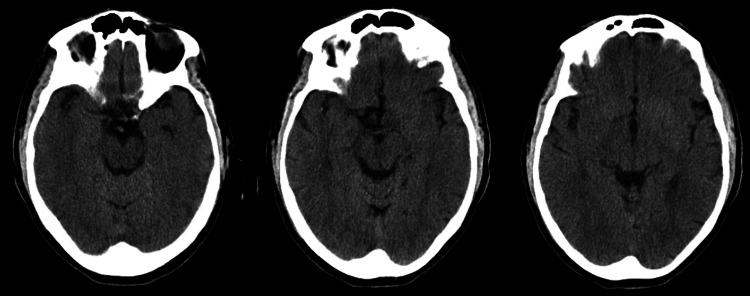
Axial non-contrast enhanced head CT showing absent hyperintensity in the temporal lobes CT: computed tomography

**Figure 2:**
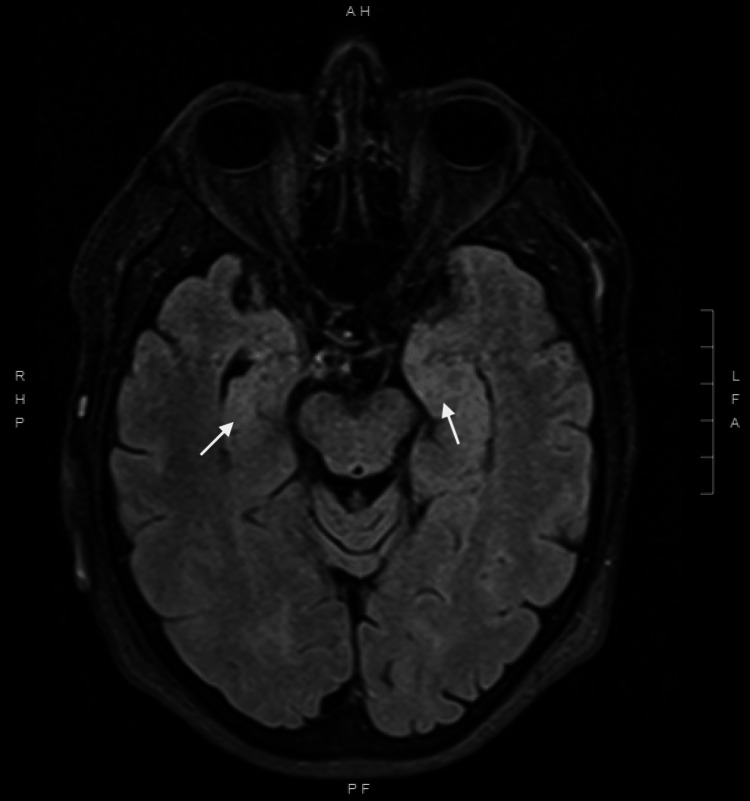
MRI brain showing mild hyperintensities isolated to the bilateral temporal lobes (arrows). These findings are consistent with limbic encephalitis, and more diffuse hyperintensities outside of the temporal lobes would be expected in viral encephalitis MRI: magnetic resonance imaging

**Figure 3:**
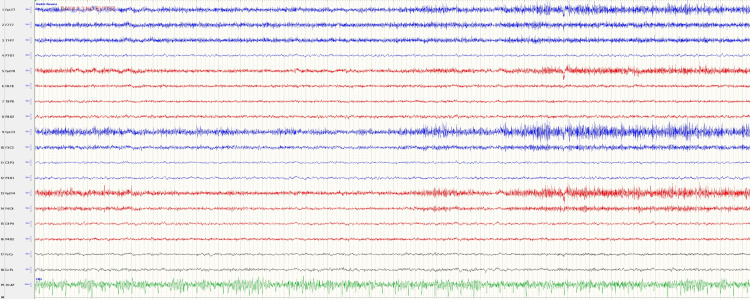
EEG obtained during an episode of abnormal facial and arm movement without any electrographic correlate, which is typical for FBDS EEG: electroencephalogram, FBDS: faciobrachial dystonic seizures

A computed tomography (CT) scan of the head was obtained and showed no significant findings (Figure 1). Magnetic resonance imaging (MRI) of the brain revealed mild hyperintense areas in the bilateral temporal lobes (Figure 2). Later, a video-EEG was performed, which demonstrated brief episodes of dystonic contraction of the face and ipsilateral arm suggestive of faciobrachial dystonic seizures (FBDS) without any electrographic correlates (Figure 3). This constellation of findings was concerning for LGI1-Ab LE. To confirm the diagnosis, a lumbar puncture was performed. CSF studies were negative for infection but also didn’t show any evidence of a CSF pleocytosis. About a week later, the CSF encephalitis panel resulted and was positive for LGI1-IgG antibodies.

Initially, he was started on IV methylprednisolone and levetiracetam for presumed LGI1-Ab LE. Given his history of weight loss, a testicular ultrasound and CT scan of the chest, abdomen, and pelvis were obtained to evaluate for a possible paraneoplastic syndrome, which would be atypical of LG1-Ab LE. These tests were negative for evidence of malignancy. Following his initial course of methylprednisolone, he was transitioned to a prolonged prednisone taper. Repeat video-EEG showed ongoing FBDS despite initial treatment. Plasmapheresis was trialed, which also failed to adequately control his symptoms. Next, he was started on lacosamide, which resulted in both subjective and EEG evidence of decreased FBDS burden. At discharge, the patient’s FBDS burden had significantly improved. He was discharged on lacosamide and a steroid taper. After discharge, he followed up in the neuroimmunology clinic, where he was started on rituximab, which resulted in the resolution of FBDS and improvement in his cognitive symptoms.

## Discussion

AMS is one of the most common chief complaints admitted to the hospital. Common differential diagnoses often include infection, electrolyte disturbances, hypoglycemia, hypoxia, substance use, and polypharmacy. It is important to keep a broad differential in mind when evaluating these patients and to complete a thorough evaluation. AE is a rare condition that can easily be overlooked. It is essential to identify atypical symptoms, such as abnormal movements, that separate AE from more common forms of AMS.

LGI1-Ab LE is the second most common cause of AE, following NMDAR-antibody encephalitis, and is estimated to have a prevalence of 0.7 cases per 100,000 persons [4]. It presents with several unique features that help to distinguish it from other types of AE. The most characteristic clinical finding is that of FBDS, which is pathognomonic for LGI1-Ab LE. These are a specific type of seizure that causes brief tonic contractions, typically of the arm and the ipsilateral face, multiple times per day [5]. These seizures typically present early in the disease course, prior to any cognitive decline, and crescendo in severity about three to six months after starting [6]. FBDS typically does not demonstrate any electrographic correlate on EEG [7].

Other common findings include bradyphrenia, which is mental slowing characterized by delayed responses and impaired attention. Bradyphrenia is not unique to AE and can be seen in other neurologic disorders such as Parkinson’s disease. Patients with LGI1-Ab LE also often exhibit short-term memory decline, behavioral disturbances, and spatial disorientation [6]. This decline in spatial orientation may be what led to the patient, in our case, having multiple car accidents prior to his presentation to the hospital.

As seen with the patient in our case, hyponatremia is a common complication of LGI1-Ab LE. About 60-88% of patients with LGI1-Ab LE will develop hyponatremia within the disease course [8]. The exact mechanism for this relationship is unknown; however, laboratory investigations typically reveal features consistent with SIADH [8]. While the hyponatremia is typically only mild to moderate in severity, it can easily be confounded as an independent cause for AMS. Hence, recognition of this relationship in patients with LGI1-Ab LE is important.

Findings on brain MRI also provide important diagnostic clues and are an essential component in the workup of LGI1-Ab LE. Not only does it help exclude competing etiologies, but it also reveals hallmark findings suspicious for underlying limbic encephalitis. Patients with LGI1-Ab LE will typically demonstrate T2 or fluid-attenuated inversion recovery (FLAIR) hyperintensities confined to the temporal lobe [9]. Other key differential diagnoses, such as viral encephalitis and Creutzfeldt-Jakob disease, typically show T2/FLAIR hyperintensities that extend beyond the temporal lobes. While the absence of T2/FLAIR hyperintensities does not exclude a diagnosis of LGI1-Ab LE, the isolation of the hyperintensities to the temporal lobe helps narrow the differential.

Establishing a diagnosis of LGI1-Ab LE can be challenging. Early diagnostic approaches to AE relied heavily on antibody testing, which is not widely available and often takes weeks to result. More recent guidelines, such as those proposed by Graus et al., focus on clinical symptoms and widely available tests such as MRI and CSF studies. For limbic encephalitis, a definite diagnosis can be made if the following criteria are met: a subacute onset (<3 months) of neuropsychiatric symptoms or seizures, bilateral abnormalities of the medial temporal lobes on MRI, CSF pleocytosis or epileptic activity involving the temporal lobe, and reasonable exclusion of other causes [10]. Our patient didn’t meet all of these criteria due to the absence of CSF pleocytosis and electrographic correlates on EEG; however, given the presence of FBDS, there was sufficient clinical suspicion to proceed with treatment.

Due to our patient’s reported weight loss, we initiated a secondary workup for malignancy to rule out a paraneoplastic process. However, LGI1-Ab LE is typically non-paraneoplastic. LGI1-Ab LE typically responds well to immunotherapy, with systemic steroids being the first-line treatment [11]. Plasma exchange, intravenous immunoglobulins, and rituximab can be used as second-line treatment options [11].

## Conclusions

This case highlights the importance of clinicians completing a thorough evaluation of all patients presenting to the hospital with AMS. AE should be considered in any case of AMS accompanied by atypical symptoms such as rapid cognitive decline, memory impairment, or abnormal movements. Recognizing FBDS and its association with LGI1-Ab LE is essential for making a quick diagnosis and initiating treatment. Hyperintensities on MRI, isolated to the temporal region, and hyponatremia can provide further diagnostic clues.
